# Spindle epithelial tumor with thymus-like elements (SETTLE): a surgical case diagnosed preoperatively using fine-needle aspiration cytology

**DOI:** 10.1530/EDM-25-0014

**Published:** 2025-05-14

**Authors:** Fumiaki Kawano, Teru Chiyotanda, Kazuhiko Nakame, Satoru Meiri, Tsuyoshi Fukushima, Kousei Shirahama, Yuichiro Sato, Hideki Yamaguchi, Makoto Ikenoue, Shun Munakata, Kazuhiro Higuchi, Shinsuke Takeno, Atsushi Nanashima

**Affiliations:** ^1^ Department of Surgery, Faculty of Medicine, University of Miyazaki, Miyazaki, Japan; ^2^ Department of Pediatrics, Faculty of Medicine, University of Miyazaki, Miyazaki, Japan; ^3^ Section of Oncopathology and Morphological Pathology, Department of Pathology, Faculty of Medicine, University of Miyazaki, Miyazaki, Japan; ^4^ Department of Diagnostic Pathology, Faculty of Medicine, University of Miyazaki, Miyazaki, Japan; ^5^ Department of Neurology, Respirology, Endocrinology and Metabolism, University of Miyazaki, Miyazaki, Japan

**Keywords:** spindle epithelial tumor with thymus-like elements (SETTLE), thyroid, fine-needle aspiration cytology

## Abstract

**Summary:**

Spindle epithelial tumor with thymic-like elements (SETTLE) is an extremely rare tumor that occurs primarily in the thyroid gland. Histologically, SETTLE is characterized by the presence of spindle-shaped epithelial cells and glandular structures. However, it is known that diagnosis via fine-needle aspiration cytology can be challenging. SETTLE predominantly occurs in younger individuals and has a less favorable prognosis compared to differentiated thyroid carcinoma. Therefore, ensuring accurate diagnosis and appropriate treatment is crucial. We encountered a case of spindle epithelial tumor with thymus-like differentiation in a 10-year-old patient for whom the preoperative diagnosis was successfully established through fine-needle aspiration cytology, which facilitated appropriate surgical resection. Comprehensive histopathological examination and immunohistochemical analysis are essential to ensure appropriate management and surveillance of SETTLE.

**Learning points:**

- A rare thyroid tumor, spindle epithelial tumor with thymic-like elements (SETTLE), was diagnosed preoperatively and treated surgically.
- SETTLE presents with characteristic histological features that must be recognized for accurate diagnosis. In addition, diagnosis through cytology is often challenging.
- The primary treatment for SETTLE is surgical intervention as radiotherapy and pharmacological treatments are generally not expected to be highly effective.
- Radical resection is the only effective treatment, making the selection of the surgical procedure according to the stage of the disease essential.

## Background

Spindle epithelial tumor with thymic-like elements (SETTLE) is an extremely rare tumor that occurs primarily in the thyroid gland. Histologically, it is characterized by the presence of spindle epithelial cells and glandular structures (1). The etiology and pathogenesis of SETTLE have not yet been fully elucidated (2). SETTLE predominantly occurs in younger individuals and has a less favorable prognosis compared to differentiated thyroid carcinoma (3, 4). Therefore, it is essential to ensure an accurate diagnosis and appropriate treatment. In addition, the difficulty of making a diagnosis through fine-needle aspiration cytology is an important consideration. In this report, we present a typical case of SETTLE, which we encountered in our clinical practice, to highlight the need for awareness of this rare tumor and the challenges involved in diagnosing it.

## Case presentation

A 10-year-old boy was referred to a primary care physician after a cervical mass was noted during a school physical examination. After examination, a thyroid tumor was suspected, and the patient was referred to our department for further examination and treatment. On physical examination, a painless, firm and solid mass was palpated on the left side of the trachea, and the tumor moved with deglutition. He did not present with hoarseness, dysphagia or any other associated symptoms. He had no developmental disabilities and no significant medical history or ongoing medical conditions.

## Investigation

**Figure 1:**
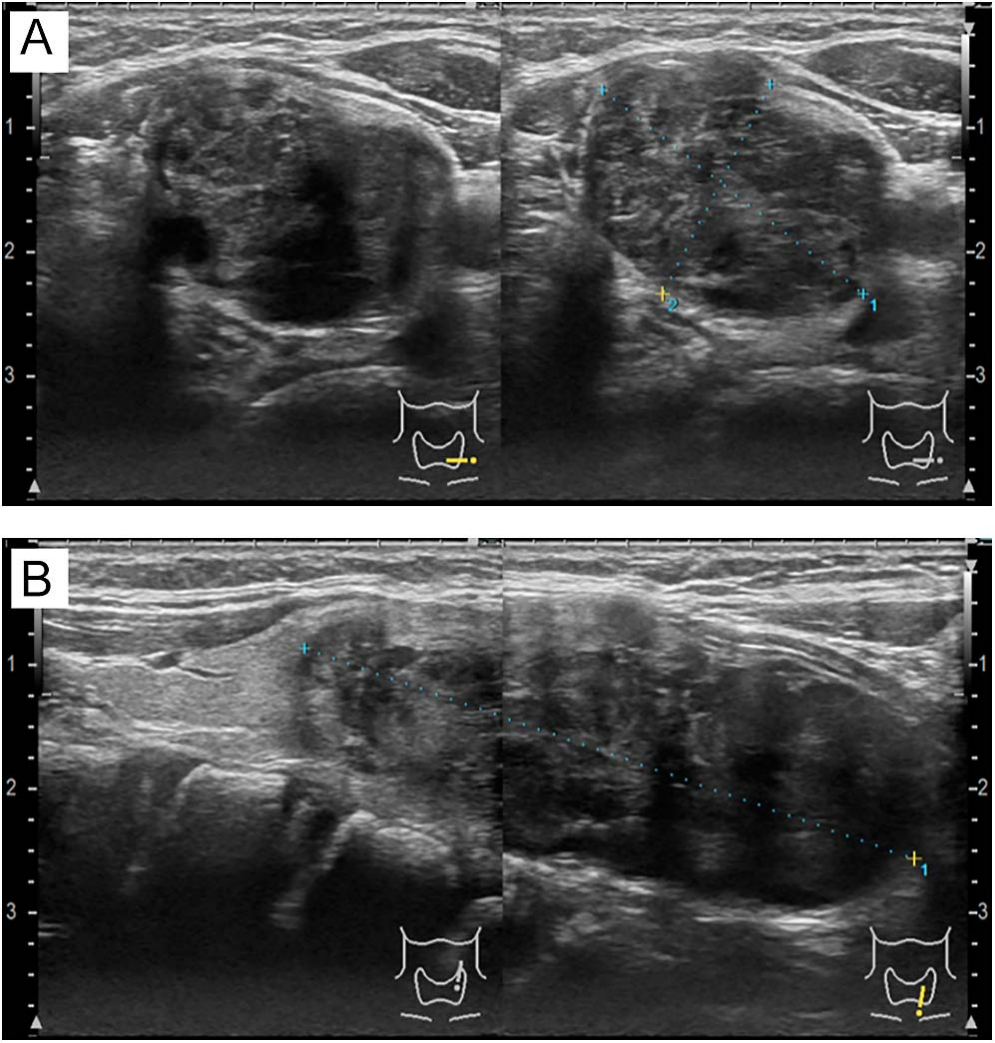
Cervical ultrasonography showed a 50 mm large heterogeneous mass extending from the left lobe of the thyroid gland to the mediastinum. (A) Transverse view. (B) Longitudinal view.

**Figure 2:**
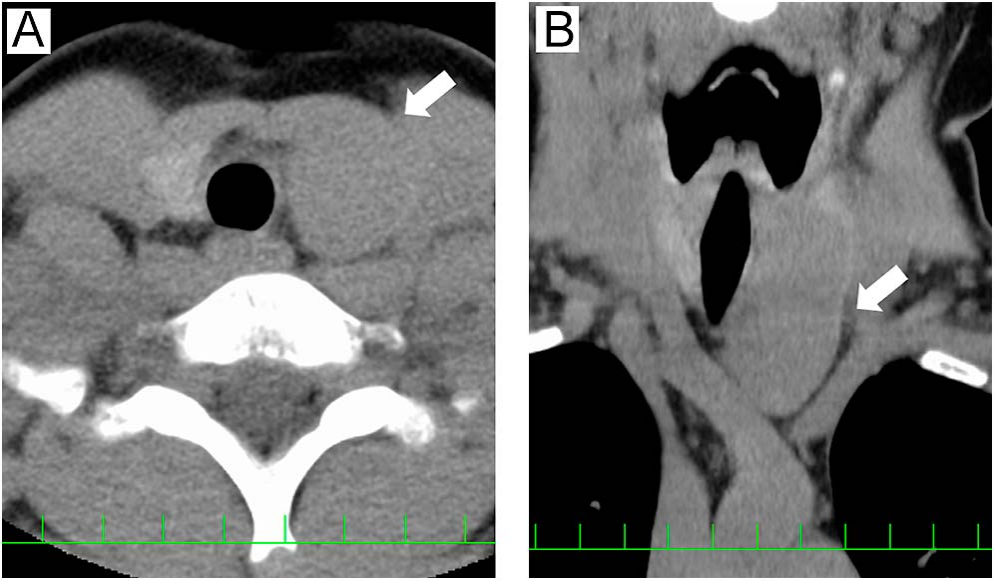
(A) CT scan showed homogeneous and hypodense mass on the left side of the trachea. (B) The lower pole of the tumor extended into the mediastinum behind the sternum.

**Figure 3:**
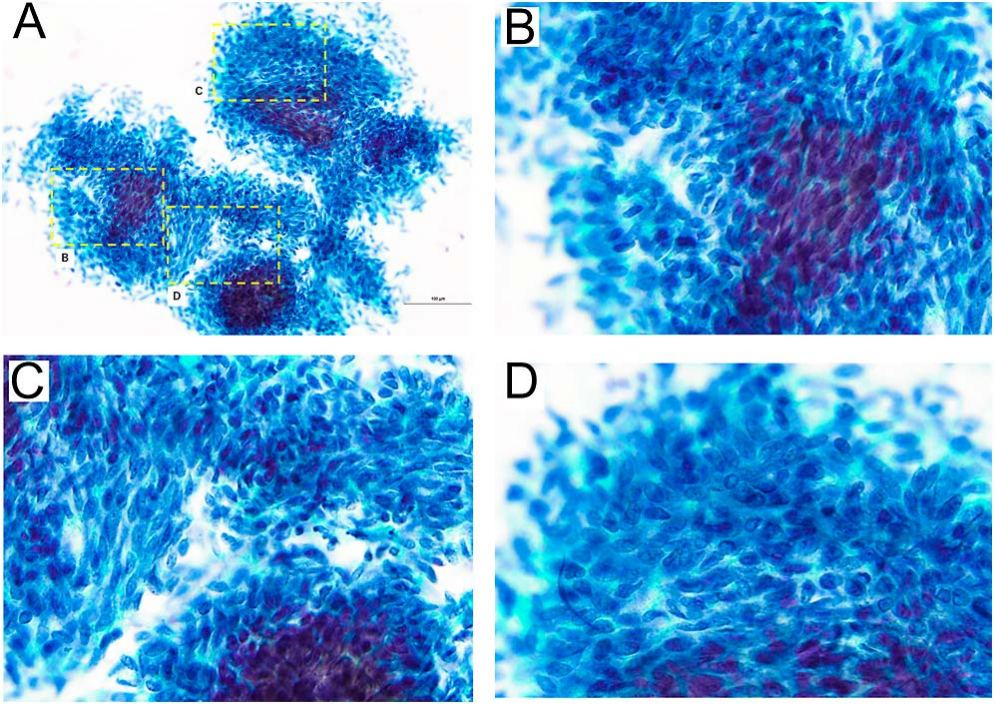
Fine-needle aspirate smears showed characteristic findings (A), including cell clusters with tubular structures (B) and spindle-shaped cell clusters (C). Some cells also showed intranuclear cytoplasmic inclusion bodies (D) that were suspicious for papillary carcinoma.

Cervical ultrasonography showed a 50 mm large heterogeneous mass extending from the left lobe of the thyroid gland to the mediastinum. The surface of the tumor exhibited a nodular appearance, whereas the internal structure displayed a mosaic pattern in some areas (Fig. 1). No abnormal findings were observed in the right lobe of the thyroid, and no lymphadenopathy was noted. Computed tomography scan revealed a 50 × 35 mm mass on the left side of the trachea. The tumor occupied the left lobe of the thyroid and extended to the upper mediastinum. There were no findings suggestive of invasion into the trachea, esophagus or the cervical and thoracic vessels (Fig. 2). He underwent fine-needle aspiration cytology, the results of which indicated abnormal findings. Tumor cells consisting of ductal structures and bundles of spindle-shaped cells appeared in clusters or agglomerations. A few intranuclear cytoplasmic inclusions were also observed. Based on the nuclear findings, a diagnosis of papillary thyroid carcinoma was considered, and the presence of spindle-shaped tumor cells raised the possibility of thyroid sarcoma. However, after comprehensive evaluation, SETTLE was the most likely diagnosis (Fig. 3).

## Treatment

**Figure 4:**
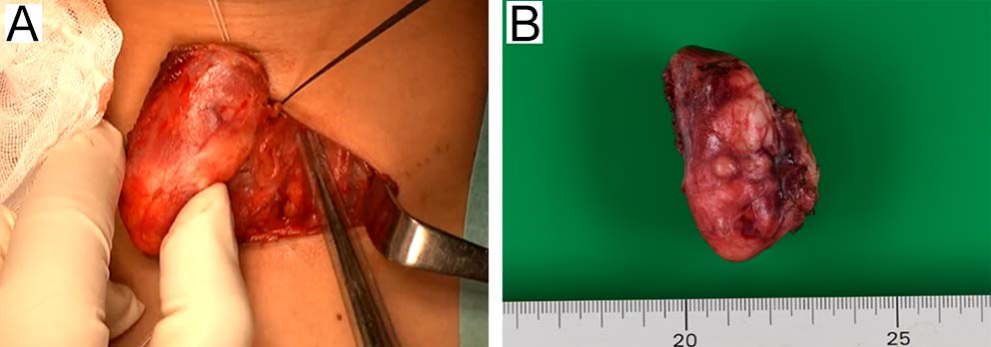
Left hemithyroidectomy were performed. (A) The tumor was easily mobilized without invasion into surrounding organs. (B) The tumor was encapsulated, exhibiting a white, smooth surface with a nodular appearance.

The patient underwent surgical resection with a clinical diagnosis of thyroid malignant tumor (suspected SETTLE or papillary carcinoma). Left hemithyroidectomy and central neck dissection were performed. The tumor was firm and white in appearance, covered by a capsule and was easily mobilized without invasion into the surrounding organs (Fig. 4). The preoperative examination showed no nodules in the right thyroid lobe. As the patient was pediatric, the right lobe was preserved to maintain thyroid function. No lymphadenopathy was found, so prophylactic central neck dissection (level 6–7) was performed.

**Figure 5:**
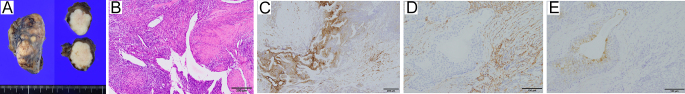
(A) Macroscopic findings of the tumor show a nodular appearance and well-defined margins. (B) Histopathological examination revealed spindle-shaped cells proliferating in an interlacing pattern and glandular structures interspersed within the spindle cell component. Immunohistochemical examination shows that the spindle tumor cells were immunoreactive to CAM (C) and vimentin (D). (E) EMA staining was negative in the spindle cells but positive in the cells of the glandular structures.

The cut surface of the tumor was white in color, with a nodular appearance and well-defined margins. This distinct demarcation suggests a clear separation from the surrounding tissues, which is characteristic of this tumor’s growth pattern. Histopathological examination revealed spindle-shaped cells with elongated nuclei proliferating in an interlacing pattern. In addition, there were occasional glandular structures interspersed within the spindle cell component. When correlated with cytological findings, these observations were consistent with a diagnosis of SETTLE. The combination of spindle cell proliferation and glandular elements supports the characteristic biphasic nature of SETTLE, aligning with its known histopathological features. The spindle tumor cells were immunoreactive to vimentin, CAM, p40, cytokeratin (CK) 7 and CD34, but negative for EMA, desmin, TTF-1 and thyroglobulin. In contrast, the glandular cells were immunoreactive to EMA. No immature lymphoid cells immunoreactive for TdT could be found. The rate of MIB-1 positivity was 10.0% (113/1,132) (Fig. 5). These histopathological findings finally led to the diagnosis of SETTLE.

The tumor showed no invasion beyond the thyroid capsule, and the surgical margins were negative. In addition, the dissected tissue did not contain any lymph nodes and was predominantly composed of thymic tissue.

## Outcome and follow-up

The patient’s postoperative course was uneventful, with no complications observed. He is currently under surveillance without any adjuvant therapy. At 12 months of postoperative follow-up, the patient has no symptoms and no findings of recurrence by physical and radiologic examination.

## Discussion

SETTLE is a rare neoplasm that primarily affects the head and neck region, particularly the thyroid gland. This tumor, named by Chan and Rosai in 1991, is recognized as a malignant tumor and is characterized by its unique histological features, which include spindle-shaped epithelial cells and the presence of thymus-like structures (5). The etiology and pathogenesis of SETTLE remain poorly understood, but its distinct morphological characteristics suggest a possible relationship to both epithelial and thymic differentiation (2). The embryonic tissues responsible for the development of the parathyroid and thymus are derived from the same origin. It has been suggested that residual thymic tissue may remain within the thyroid and parathyroid glands during the formation of the thyroid gland. This retained thymic tissue can potentially undergo tumorigenic transformation, leading to the development of tumors (3).

This tumor is commonly observed in children and adolescents, with very few reports in adults. It tends to occur more frequently in males, and the size of the tumor is generally considered to be larger than that of typical thyroid tumors (2, 4, 6). The clinical presentation of SETTLE is often perceived as painless neck masses; however, they may occasionally present with compressive symptoms such as dysphagia or superior vena cava syndrome (3, 6, 7). Ultrasonography often shows a hypoechoic or heterogeneous solid pattern, but these findings are not characteristic (6). In this way, imaging studies typically reveal a well-defined mass, which can sometimes be mistaken for more common thyroid lesions, such as follicular adenomas or carcinomas. In many cases, blood tests do not show abnormalities, making it difficult to suspect this disease based on any of the test findings.

The primary treatment is surgical resection, with most reported cases undergoing surgery. Depending on the extent of the tumor and the institution’s policy, either total thyroidectomy or lobectomy is selected (4, 6). Lymph node dissection is performed if lymph node metastasis or metastases are suspected. There are reports of good outcomes with aggressive surgical resection even in distant metastatic sites where surgical intervention is feasible (2). SETTLE should be considered for aggressive intervention aimed at radical resection as it is not expected to respond at all to radioactive iodine therapy. The effectiveness of radiotherapy and chemotherapy has also been reported in unresectable cases and those with distant metastases (3, 4). Due to its rarity, the optimal management of this condition and the role of adjuvant therapies, such as chemotherapy and radiotherapy, remain subjects of ongoing debate. This variability in response could be attributed to the tumor’s unique histological characteristics and the lack of large, controlled studies evaluating the efficacy of chemotherapeutic agents. In instances where surgical resection is not feasible, or residual disease is present, radiotherapy has shown some potential in controlling local disease progression (4). Nevertheless, its impact on overall survival remains unclear. Until more robust evidence is available, treatment should be tailored to the individual patient, carefully balancing the potential benefits of adjuvant therapies against their associated side effects. SETTLE is reported to have a higher risk of recurrence and mortality compared to papillary thyroid carcinoma (3, 4). Long-term monitoring is essential given the tumor’s tendency for late recurrence and metastasis. Regular imaging and clinical assessments are recommended to promptly detect any signs of metastatic disease.

SETTLE is histologically characterized by a biphasic pattern, consisting of two distinct components, the first of which is composed of spindle-shaped cells that are arranged in fascicles or bundles. These spindle cells exhibit a fibrous or fascicular growth pattern, often showing mild nuclear atypia and scant cytoplasm. The second component consists of cuboidal to columnar epithelial cells that form glandular or tubular structures. These epithelial cells contribute to the thymus-like differentiation, a hallmark feature of SETTLE. The dual presence of spindle cell fascicles and glandular epithelial elements is a defining characteristic, aiding in the histopathological identification of this rare tumor. Immunostaining is essential for the histopathological diagnosis of this tumor. Both spindle cells and tubular cells show positive staining for CK, including CK7. The spindle cell component may also show reactivity for vimentin, EMA, CD99, CD117, TLE1 and myoepithelial markers such as SMA and MSA. No reactivity is observed for CK20, thyroglobulin, calcitonin, CEA, TTF-1, CD5, TdT, S-100 protein, desmin, CD34, calretinin or WT-1. In rare cases, squamous epithelium may also be identified. The absence of SS18 gene translocation is a key feature that helps distinguish this tumor from synovial sarcoma (8, 9).

The diagnosis of SETTLE is challenging using fine-needle aspiration cytology (1, 2). This difficulty primarily arises from the tumor’s unique histological features, which may not be adequately represented in cytological specimens. Fine-needle aspiration samples typically contain only a limited number of cells, making it difficult to identify the biphasic nature of SETTLE, which includes both spindle cells and epithelial components. In addition, because SETTLE is a very rare tumor, many cytologists may lack experience in evaluating such cases. While immunocytochemistry can assist in distinguishing SETTLE from other spindle cell tumors, this adjunct test may not be sufficient for diagnosis if the cytological sample does not capture both the spindle cell and epithelial components. Therefore, a more extensive tissue sample, such as from a surgical resection, may be necessary to obtain the required histopathological context for an accurate diagnosis of SETTLE. To achieve a diagnosis through cytology, it may be crucial to obtain an adequate cellular sample and to be aware of the possibility of the existence of SETTLE. To obtain an adequate tissue sample, we performed a total of four fine-needle aspiration biopsies with the patient under sedation. This approach may have played a key role in establishing the preoperative diagnosis. In this case, the strong preoperative suspicion of SETTLE allowed us to anticipate the extent of resection and the scope of lymph node dissection, based on a comprehensive understanding of the disease’s pathophysiology, treatment, and prognosis. Furthermore, before surgery, the patient’s family was thoroughly informed about the rarity of the condition, and their understanding and consent were successfully obtained.

Fine-needle aspiration for the diagnosis of pediatric thyroid tumors has been routinely performed at our institution under sedation in the outpatient procedure room, with pediatrician supervision. In this case, given the specific imaging findings and the necessity for obtaining an adequate tissue sample for diagnosis, along with the patient’s significant anxiety and the family’s request, FNA was performed under sedation with intravenous administration of 0.25 g thiamylal sodium. During the procedure, there were no signs of patient movement, and the procedure was safely conducted. The patient rapidly regained consciousness and was able to walk and return home shortly thereafter. Mazzantini *et al.* reported that FNA of pediatric thyroid lesions can be performed safely and with high diagnostic accuracy even without sedation (10). Therefore, it is anticipated that, as the number of pediatric FNA cases increases and operators gain more experience, non-sedated FNA may become more common in our institution in the future.

## Conclusion

SETTLE is a rare thyroid malignancy that predominantly affects young individuals. SETTLE has the potential for late metastasis, underscoring the importance of accurate diagnosis and long-term follow-up. Fine-needle aspiration cytology often poses diagnostic challenges due to the tumor’s biphasic nature and rarity. Therefore, clinicians and pathologists should maintain a high index of suspicion for SETTLE when evaluating biphasic thyroid tumors and consider comprehensive histopathological examination and immunohistochemical analysis to ensure appropriate management and surveillance.

## Declaration of interest

The authors declare that there are no conflicts of interest that could be perceived as prejudicing the impartiality of the work reported.

## Funding

This work did not receive any specific grant from any funding agency in the public, commercial or not-for-profit sector.

## Patient consent

Written informed consent for publication of the clinical details and images was obtained from the patient’s parents.

## Author contribution statement

F Kawano, H Yamaguchi and A Nanashima wrote the case report. T Fukushima, K Shirahama and Y Sato prepared and commented on the pathological findings. T Chiyotanda, K Nakame, S Meiri, M Ikenoue, S Munakata, K Higuchi and S Takeno contributed to the clinical management of this patient.
